# Neutrophil extracellular traps induce the bone erosion of gout

**DOI:** 10.1186/s12891-022-06115-w

**Published:** 2022-12-26

**Authors:** Ertao Jia, Zhiling Li, Hongling Geng, Haiqiong Zhu, Yadong Wang, Feng Lin, Yubao Jiang, Jianyong Zhang

**Affiliations:** 1 The Department of Rheumatology, the Fourth Clinical Medical College of Guangzhou University of Chinese Medicine, No.1, Fuhua Road, Futian District, Shenzhen, 518033 Guangdong China; 2 The Department of Rheumatology, Shenzhen Traditional Chinese Medicine Hospital, Shenzhen, China; 3 The Department of Rheumatology, Shenzhen Traditional Chinese Medicine Hospital; and the Fourth Clinical Medical College of Guangzhou University of Chinese Medicine, No.1, Fuhua Road, Futian District, Shenzhen, 518033 Guangdong China; 4 The Fourth Clinical Medical College of Guangzhou University of Chinese Medicine, Shenzhen, China; 5 The Department of Gynecology, Guangdong Provincial Hospital of Chinese Medicine, the Second Affiliated Hospital of Guangzhou University of Chinese Medicine, Guangzhou, China; 6 Shenzhen Traditional Chinese Medicine Hospital Affiliated to Nanjing University of Chinese Medicine, Shenzhen, China; 7 The Department of Urology, Shenzhen Traditional Chinese Medicine Hospital, Shenzhen, China

**Keywords:** Gout, Neutrophil extracellular traps, Osteoblasts, Osteoclasts

## Abstract

**Objective:**

To investigate the relationships between monosodium urate (MSU) crystals -induced neutrophil extracellular traps (NETs) and bone erosion in gout.

**Methods:**

Animal models were used to study the relationship between NETs induced by MSU crystals and bone erosion. Neutrophils were treated with MSU crystals to induce NETs. The osteoblasts-like cells (OB) were then treated with NETs, and the supernatant was co-incubated with osteoclasts-like cells (OC). The NETs were digested with DNase, and the neutrophil elastase (NE) was inhibited with sivelestat sodium. Cell viability, mRNA, and protein expression were also assessed.

**Results:**

After treating OB with NETs, the cell viability decreased. Yet, after digesting the DNA and inhibiting NE, the viability was moderately improved. The expression level of osteoprotegerin (OPG) and alkaline phosphatase (ALP) was up-regulated, while the expression level of receptor activator of nuclear factor kappa-B ligand (RANKL) was down-regulated in the sivelestat sodium + MSU group compared with MSU group. The number of OC was significantly elevated. In contrast, the number of OB was not increased in the tibia after establishing the gout model. The supernatant obtained from OB was treated with NETs promoting OC differentiation. The expression level of receptor activator of nuclear factor kappa-B (RANK), tartrate-resistant acid phosphatase (TRAP), and cathepsin K (Cst K) was up-regulated in the MSU group compared with the normal control (NC) group.

**Conclusion:**

NETs induced by MSU crystals could inhibit osteoblasts viability and enhance the activity of osteoclasts.

**Supplementary Information:**

The online version contains supplementary material available at 10.1186/s12891-022-06115-w.

## Introduction

Gout is a common arthritic condition caused by the deposition of monosodium urate (MSU) crystals. In 2015–2016, the overall prevalence of gout among American adults was approximately 3.9% [1]. Joint damage is frequently observed in patients with advanced gout, and bone erosion and new bone formation (NBF) may occur in affected joints [2]. Imaging studies have suggested that tophus has an important role in developing bone erosion and NBF [3, 4]. In addition, dual-energy computed tomography (CT) studies have demonstrated a close relationship between tophus and bone erosion [5]. Furthermore, a magnetic resonance imaging (MRI) study indicated that cartilage damage is not associated with bone edema (BME) in gout patients [6].

Osteoprotegerin (OPG)/receptor activator of the nuclear factor kappa-B (RANK) pathway has a crucial role in the joint damage of gout [7]. However, the mediators of tophus that act on the osteoblasts (OB) and osteoclasts (OC) remain unclear. Some studies have found that the MSU crystals are entrapped by neutrophil extracellular traps (NETs) [8, 9], which are composed of extracellular DNA networks associated with histones and polymorphonuclear neutrophils granule proteins, including myeloperoxidase (MPO) and neutrophil elastase (NE) [10]. NETs have a diverse role in different diseases [11], and NE, which comes from the cytoplasm, is the main activity cluster of NETs [12]. NE can degrade components, such as cytokines [13]. This study investigated the relationships between MSU crystals-induced NETs and bone erosion in gout.

## Materials and methods

### Animals

Male Sprague–Dawley (SD) rats (8-week-old) were obtained from Guangdong medical laboratory animal center. Rats were randomly assigned to NC and MSU groups (three rats per group). The necessary sample sizes were biometrically estimated. All the animals were housed in an environment with a temperature of 22 ± 1 ºC, relative humidity of 50 ± 1%, and a light/dark cycle of 12/12 h. All animal studies (including the mice euthanasia procedure) were done in compliance with the regulations and guidelines of Guangzhou University institutional animal care and conducted according to the AAALAC and the IACUC guidelines (20211201006).

### Rat model

The rat model of gouty arthritis was established as previously described [14]. Briefly, adaptive feeding was applied for one week. Rats in the MSU group were injected with 0.2 mL of 25 mg/mL MSU crystals solution into the right ankle joint cavity, while the NC group received the same volume of PBS at the same site. The rats were sacrificed at 24 h, 7 d, and 14 d, after which the joint cavity was exposed, dissected, and rinsed three times with 3 ml PBS (supplement Fig. 1).

### Human neutrophil isolation

This study was approved by the Institutional Medical Ethics Committee of the Fourth Clinical Medical College of Guangzhou University of Chinese Medicine (K2021-082–01). The peripheral blood analyses of normal healthy donors (NHDs) were performed in accordance with institutional guidelines. Neutrophils and peripheral blood mononuclear cells (PBMCs) were isolated from the heparinized blood of NHDs by routine Ficoll density gradient centrifugation using standard protocols. In three independent experiments, the experiment was performed in triplicate with the same donor.

A total of 15 ml of human peripheral blood was collected and separated with Ficoll lymphocyte separation solution. Thereafter, the blood was centrifuged at 2000 rpm for 23 min to push the neutrophils into the upper layer of red blood cells. The erythrocyte lysate was then added at 1:3 for 20 min, centrifuged again at 1800 rpm for 5 min, and the concentration of neutrophils was measured.

### NETs formation and collection

The neutrophils were incubated in a 6-well plate at 1 × 10^6^ cells ml^−1^ and cultured for 30 min. After stimulating with MSU crystals (200 μg/ml) or PBS for 4 h at 37 ℃, the bottom of the plate was washed gently with cold PBS, and 1 ml preheated phenol red-free 1640 medium was added to the dish. Next, the bottom of the plate was repeatedly blown with a pipette to promote the dissolution of NETs into the liquid. Then, the liquid was centrifuged at 350 g, 4℃ for 10 min, and the NETs were collected.

### Cell culture

SV40 transfected human osteoblasts (hFOB 1.19) were kindly provided by Guangzhou saiku biotechnology Osteoblastic Co.Ltd. hFOB resuscitation. Cells were passaged several times and then divided into 5 groups: (1) NC Group: the supernatants obtained from neutrophils were treated with PBS; (2) MSU Group: the supernatants obtained from neutrophils were treated with MSU crystals (200 μg/ml); (3) MSU + DNase Group: the supernatants obtained from neutrophils were treated with MSU crystals (200 μg/ml) and with specific DNA endonuclease DNase for 30 min; (4) MSU + Sivelestat Group: MSU crystals (200 μg/ml) for 30 min followed by addition of 44 nM Sivelestat sodium; (5) Sivelestat + MSU Group: 44 nM sivelestat sodium for 30 min followed by addition of MSU crystals (200 μg/ml).

The method of OC formation induced by THP-1 cells kindly provided by Procell life science and technology Co., Ltd, was performed as previously described [15]. THP-1 cells were incubated with 100 ng/ml PMA for 48 h to induce osteoclast progenitor cells. Then, cells were cultured at 37 °C in the presence of 5% CO_2_; the media were replaced every three days. In order to further determine whether NETs affect osteoclast differentiation through OB, the osteoclast progenitor cells induced by THP-1 cells were co-cultured with the supernatant obtained in the previous step (NC group, MSU group) for 24 h.

### Preparation of the MSU

MSU crystal was prepared following the protocol [16].

### Cell Counting Kit-8 Assay

CCK-8 assay was used to detect the OB cell viability and proliferation. Briefly, the cells were cultured with different supernatants for 48 h. A 3 × 10^4^ cells/ml were then added into 96-well plates (100 μl per well) and incubated at 37℃ under water-saturated 95% air and 5% CO_2_ atmosphere. After 24 h, 10 μL of CCK-8 reagent was added into 100 μL medium in each well, and the optical density (OD) was measured at a wavelength of 450 nm every 30 min until the OD was 1.0–2.0. The experiment was run in triplicate.

### For immunofluorescence (IF) assay

The joint cavity was repeatedly rinsed with 1 ml HANK's containing 0.1%EDTA and cytospin to collect fluid. Samples were then dried at room temperature for 6–12 h, fixed with 4% paraformaldehyde, and then incubated with 0.2% Triton X-100 for permeabilization at room temperature. Then, samples were incubated with an antibody cocktail (final concentration: anti-MPO 1:50, anti-Histone H3 1:200) overnight at 4 °C, washed with PBST 3 times before being subjected to secondary antibodies for 1 h at room temperature. Finally, samples were washed with PBST 3 times, stained with DAPI, and analyzed.

### For Hematoxylin–eosin (HE) assay

The tibia was fixed in formalin, decalcified in 10% ethylenediamine tetraacetic acid solution (EDTA, pH 7.4), embedded in paraffin, and sliced at 3 μm for hematoxylin–eosin (HE) staining. The stained tibia was imaged with a light microscope.

### For immunohistochemistry (IHC) assay

The appropriate antibody diluent and antigen repair treatment methods were selected and performed following the manufacturer’s instructions. The paraffin-embedded tissue was dewaxed in xylene, dehydrated in ethanol, and blocked in 3% H_2_O_2_ for 5–10 min. The antigen was extracted in 95 °C EDTA buffer (0.01 M, pH 6.0) for 10–15 min. Sections were then incubated with anti-neutrophil elastase (ab68672, Abcam) overnight at 4 °C. After washing three times with PBS, samples were incubated with the secondary antibody coupled to HRP at 37 °C for 50 min at room temperature. Finally, DAB chromogen was added, and slices were washed with water and stained with hematoxylin.

### For TRAP (Tartrate-Resistant Acid Phosphatase) assay

Tibia samples were embedded in paraffin and stained using the TRAP staining kit (Servicebio, China) according to the manufacturer’s instructions. OC was defined as TRAP-positive multi-nucleated cells. The cytoplasm was colored in wine red, and the nucleus in light blue.

### Alkaline phosphatase (ALP) staining and activity assay

The tissue was fixed in 4% formalin for 10 min and washed thrice with PBS. According to the manufacturer's instructions, ALP staining and activity were performed with a staining kit (Solarbio, G1480).

### Western Blot

Total proteins in tissues or cells were extracted with RIPA lysate buffer after adding a protease inhibitor cocktail. SDS-PAGE was applied to isolate equal amounts of protein, after which samples were transferred to the PVDF membrane. Consequently, the membrane was incubated with the primary antibody GAPDH (1:8000), ALP (1:5000), OPG (1:500), RANKL (1:500), TRAP (1:5000), Ctsk (1:500), RANK (1:1000), overnight at 4 °C and with HRP coupled secondary antibody for 1 h at room temperature. Anti-GAPDH antibody was utilized as an internal control. Protein expression levels were measured using an ECL substrate.

### Quantitative real-time PCR

Total RNA from each sample was extracted with Trizol (MRC, TR118-500) and then reverse-transcribed using the M-MLV Reverse Transcriptase (Promega, M1705). Amplification was performed using the GoTaq® qPCR Master Mix (Promega, A6002) with 40 cycles at 95 °C for 15 s and 60 °C for 1 min on a StepOnePlus Real-Time PCR System. Table S1 lists the primers used in this study.

### ELISA

The levels of IL-1β(Dakewe Biotech, 1310122), TNF-α(Dakewe Biotech, 1317202), NE (Cusabio, CSB-E08847r), and MPO (Cusabio, CSB-E08722R) were detected using ELISA kit according to the manufacturer's protocol. Absorbance was measured at 450 nm.

### Statistical analysis

Statistical analysis was performed with Graphpad Prism 9.0 Software. Results are presented as means ± SD of at least three independent experiments. Statistical analysis was performed using a t-test and one-way ANOVA. *p ≤ 0.05, **p ≤ 0.01 and ***p ≤ 0.001 were considered statistically significant.

## Results

### MSU crystals induce the formation of NETs in vivo

**Fig. 1:**
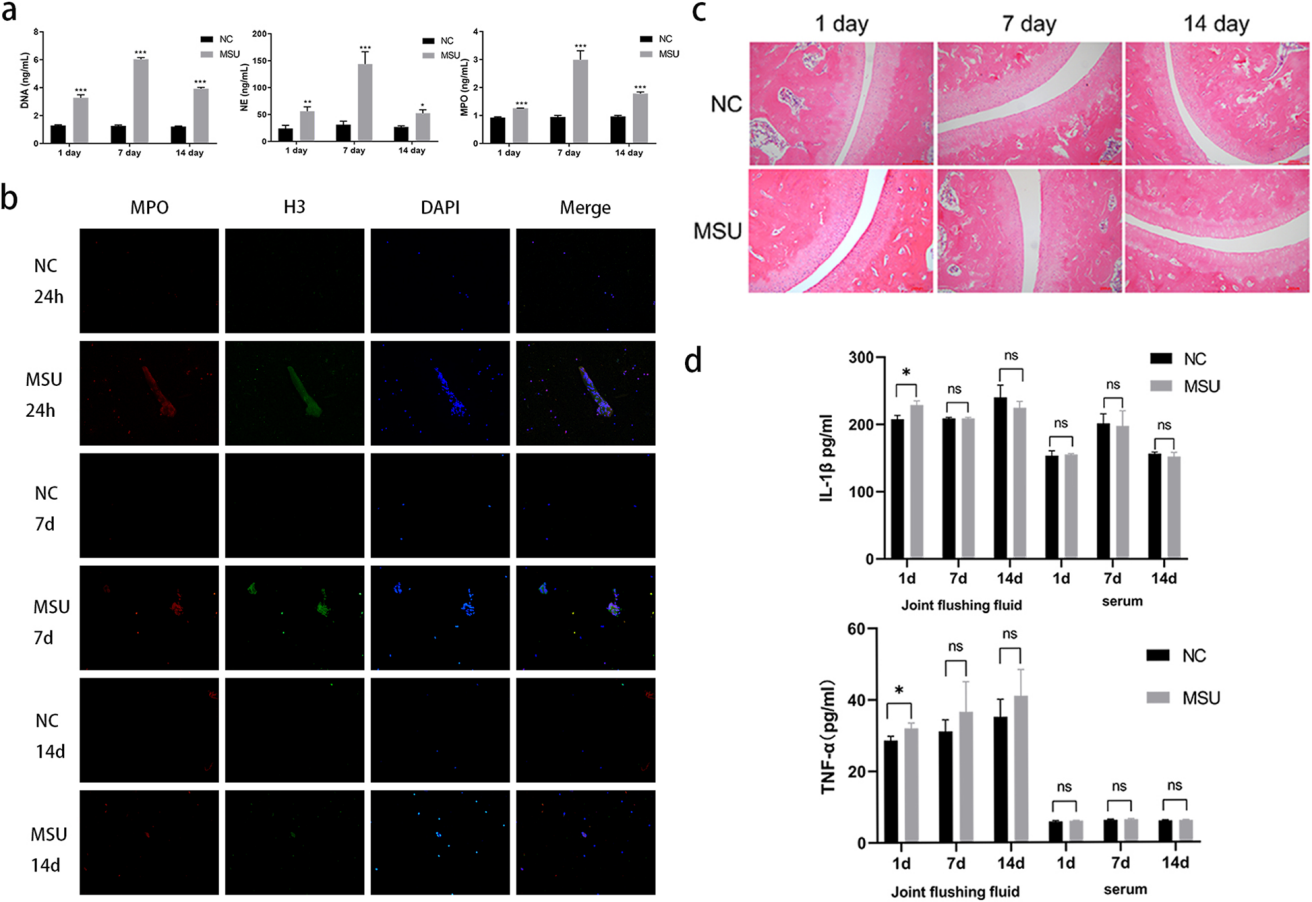
MSU crystals induce the formation of NETs in vivo. **a** The concentration of DNA, NE, MPO in the MSU group was higher than the NC group on days 1, 7 and 14. *P < 0.05, **P < 0.01, ***P < 0.001 as determined by t test (*n* = 3). **b** The immunofluorescence of the joint flushing fluid (× 20) The formation of NETs (MPO^+^H3^+^DNA^+^) in group MSU was significantly higher than NC groups on days 1, 7 and 14. **c** The histopathology of tibia (× 100). There was slight inflammatory infiltration in the cartilage on 1 day, while that was relieved at 7 day and 14 days in the MSU group. **d** IL-1β、TNF-αwere measured with ELISA. The level of two of them were higher than NC group at 24 h, on the day 7 and 14, the comparison was no longer statistically significant. This result was consistent with **c**

We found that the concentrations of DNA, NE, and MPO of the joint fluids (Fig. 1a) in the MSU group were more pronounced than in the NC group; these data were further confirmed after analyzing the joint cavity 24 h, 7 days, and 14 days after injection in vivo (Fig. 1b). The histopathology of tibia showed that the cartilage surface was smooth, the cartilage cells were regular, and there was no inflammatory cell infiltration in the cartilage and the bone in the NC group. However, in the MSU group, a small amount of inflammatory cell infiltration in the cartilage was observed after 24 h, which was significantly relieved on day 7 and 14 (Fig. 1c).

Then, we tested the level of IL-1βand TNF-α in joint flushing fluid and serum. The levels of IL-1βand TNF-αwere significantly higher in the MSU group than in the NC group at 24 h (228.8 ± 3.19 pg/ml vs. 206.0 ± 2.38 pg/ml, *p* = 0.001), and TNF-α(32.02 ± 0.75 pg/ml vs. 28.64 ± 0.59 pg/ml, *p* = 0.012); yet, no significant difference was detected on day 7 and day 14. Moreover, there were no differences in IL-1β and TNF-α levels in serum (Fig. 1d).

### MSU crystals induce the differentiation of osteoclasts in vivo

**Fig. 2:**
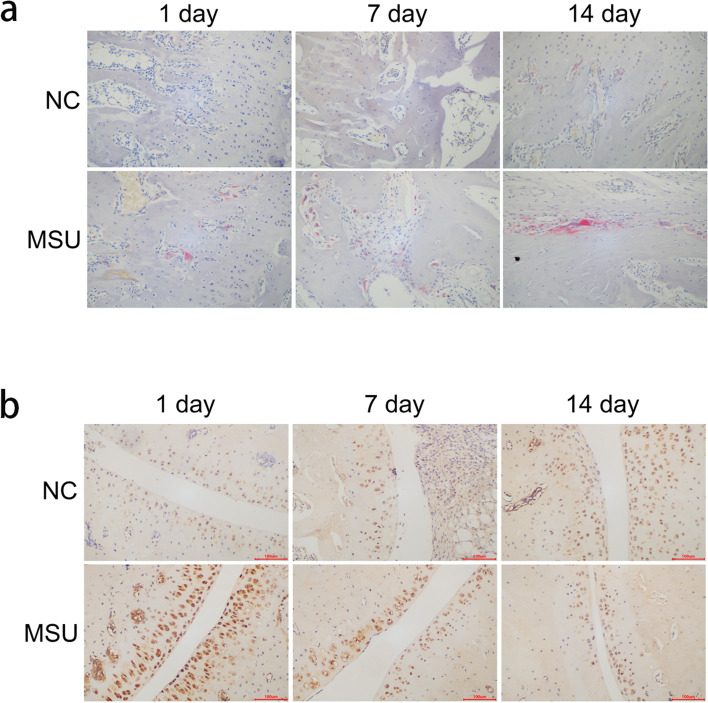
MSU crystals induce the differentiation of osteoclasts in vivo. **a** The number of osteoclasts (red by TRAP staining) was significantly increased on day 1, 7 and 14 than NC group. **b** The immunohistochemistry of the tibia (× 200). The NE expression of MSU group was significantly higher than NC group on 1 day, but there was no difference on 14th day

The TRAP staining was used to test the differentiation of the OC in the tibia. We found that the number of OC was significantly increased on day 1, 7, and 14 in the MSU group (Fig. 2a). We then performed immunohistochemistry to test the expression of NETs in the chondrocytes. The expression of NE was significantly higher in the MSU group than in the NC group on 1 day (11.74 ± 4.72% vs. 3.57 ± 1.04%, *p* = 0.007), while there was no difference on the 7^th^ day (3.83 ± 1.75% vs. 2.38 ± 0.75%, *p* = 0.643) and 14^th^ day (2.06 ± 0.57% vs. 3.05 ± 0.28%, *p* = 0.785) between MSU and NC group (Fig. 2b).

### NETs affects viability of osteoblasts in vitro

**Fig. 3:**
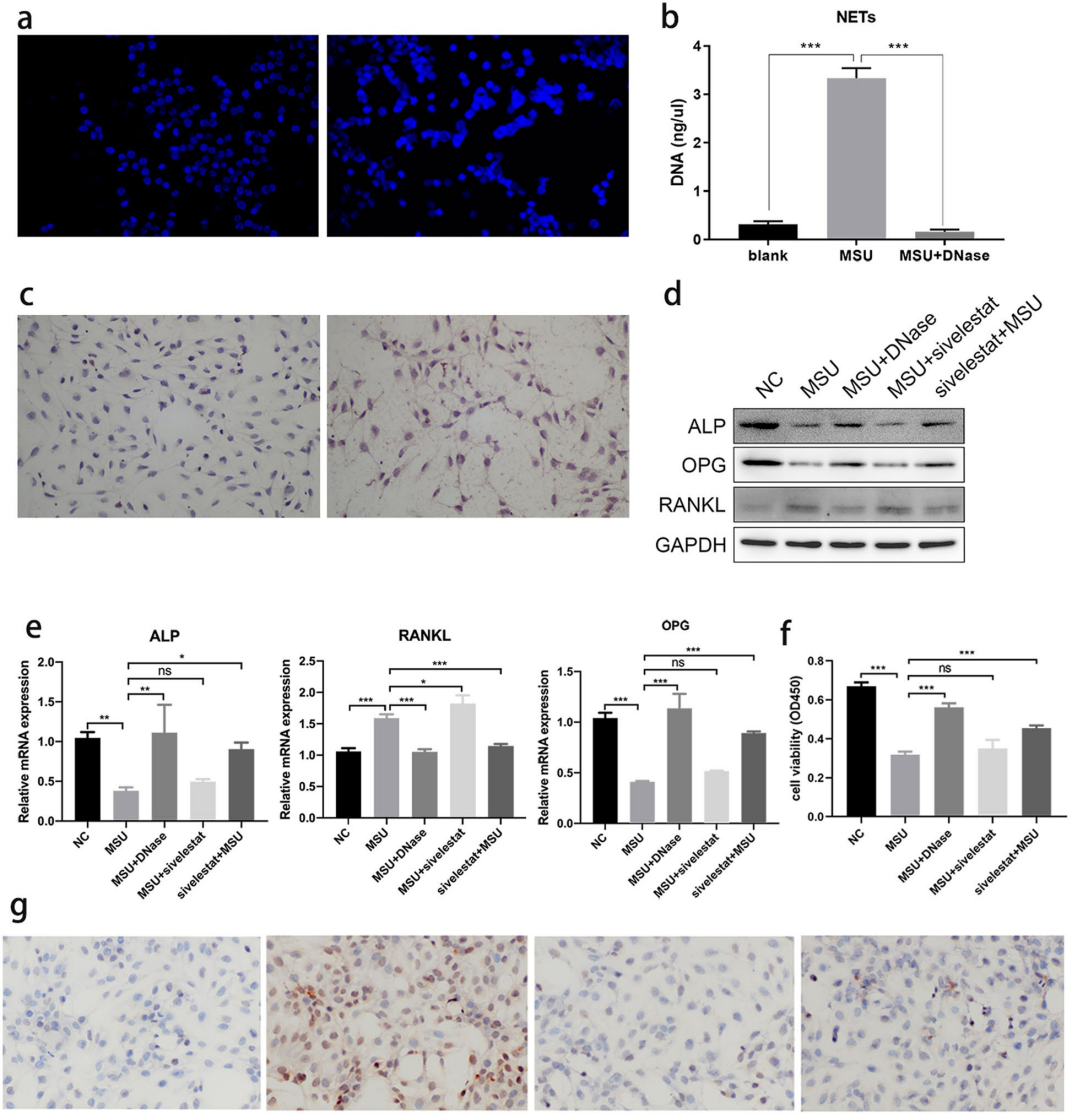
NETs affects viability of osteoblasts in vitro. The alkaline phosphatase staining of the osteoblasts (× 200). **a** MSU crystals induces the formation of NETs in vitro (× 40). DAPI fluorescence intensity and filamentous structures were observed in the MSU group. **b** The concentration of DNA was significantly higher in the MSU group than the NC group, but after the addition of DNase treatment, the supernatant DNA concentration decreased. ***P < 0.001 as determined by t test. **c** NETs adhere to osteoblasts. Osteoblasts were covered by NETs (reddish brown). **d** The expression of ALP and OPG was decreased, and RANKL was increased which were tested by WB. After digesting the NETs in the supernatant or adding the inhibitor of NE, the expression of markers was restored. **e** The ALP and OPG mRNA were decreased, while the RANKL mRNA was increased. After digesting the NETs in the supernatant or adding the inhibitor of NE, the expression of markers was restored. *P < 0.05, **P < 0.01, ***P < 0.001 as determined by t test. **f** The viability of osteoblasts was tested by CCK-8. After treating osteoblasts with supernatant containing NETs, the viability of osteoblasts decreased. After digesting the NETs in the supernatant or adding the inhibitor of NE, the viability was restored. **g** IHC showed the expression of NE increased significantly after stimulation

Neutrophils (1 × 10^6^ cells/ml) were treated with PBS and MSU crystals for 4 h, respectively. DAPI fluorescence intensity and filamentous structures were observed, which are typical for NETs in the MSU group (Fig. 3a). The concentration of DNA was significantly higher in the MSU group (3.33 ± 0.21 ng/uL) than in the NC group (0.31 ± 0.66 ng/uL), but after DNase treatment, the supernatant DNA concentration decreased (0.16 ± 0.05 ng/uL) (Fig. 3b).

The supernatant containing NETs was co-cultured OB for 48 h, NETs could coat the OB (Fig. 3c). NETs have a diverse role in different diseases [11]. A previous study found that NE is the main activity cluster of NETs [12]. Therefore, we treated the neutrophils with 44 nM sivelestat sodium, a competitive inhibitor of NE, for 30 min before and after treatment with MSU crystals [17, 18], and the supernatant was co-cultured with OB. We found that adding Sivelestat sodium first rather than later could affect the osteoblasts, up-regulated the expression level of ALP and OPG, and down-regulated the expression of RANKL in osteoclast differentiation, compared with the MSU group (Fig. 3d and e). Next, we tested the effect of NETs on the viability of OB. The viability of OB decreased after treatment with supernatant containing NETs in the MSU group. After digesting the NETs with DNase, the viability of the OB was restored (Fig. 3f).

IHC was performed to detect the expression of NE. We found that the content of NE was higher in the MSU group than in the NC and MSU + DNase groups. The content was obviously decreased in the Sivelestat + MSU group (Fig. 3g).

### NETs induce osteoclast differentiation by acting on osteoblasts in vitro

**Fig. 4:**
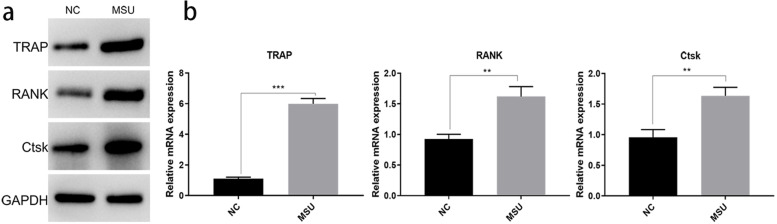
NETs induce osteoclast differentiation by acting on osteoblasts in vitro. **a** The osteoclasts differentiation in vitro. The expression of TRAP, RANK and CST were significantly increased in MSU group by Western Blot. *P < 0.05, **P < 0.01, ***P < 0.001 as determined by t test. **b** The TRAP, RANK and CST mRNA were significantly increased in MSU group

**Fig. 5:**
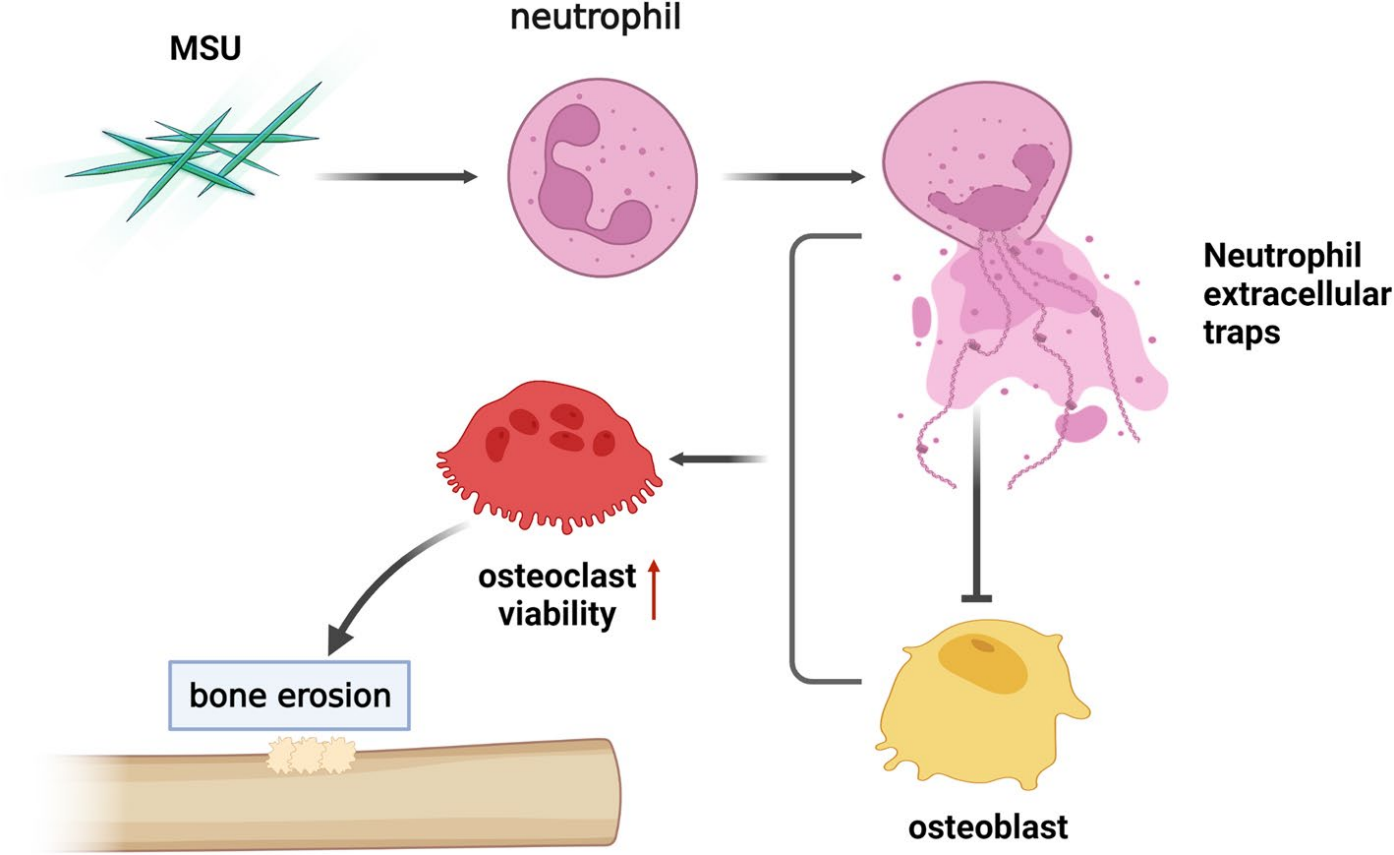
Schematic diagram of the study: Osteoblast co-incubation with NETs induced by MSU enhance the activity of osteoclast

The supernatant containing NETs was directly incubated with OC, the differentiation and activity of OC was unchanged. As far as we know, RANKL is related to OC differentiation. OB co-cultured with NETs for 48 h, and the supernatants were treated with the OC for 24 h. The markers of osteoclast differentiation, such as TRAP, RANK, and Ctsk, were significantly increased in the MSU group, which were tested by WB (Fig. 4a) and PCR (Fig. 4b). This suggested that NETs not only directly acted on OB and inhibited their viability, but also stimulated OC differentiation (Fig. 5).

## Discussion

Previous studies shown that IL-1β and TNF-αhave an important role in bone erosion of gout [7, 19]. However, gout is a typically self-limiting disease [20], different from rheumatoid arthritis, caused by bone destruction and inflammation [21]. Our study showed that levels of IL-1β and TNF-αwere not significantly increased in the joint flushing fluid of rats, whereas the presence of NETs could be detected at 7 days and 14 days. Therefore, these data suggest that NETs might participate in the pathogenesis of bone erosion in gout.

A previous study found that NETs triggered by MSU crystals can cause local cartilage damage, periphery tissue injury, and remodeling [22, 23]. However, the current study showed decreased viability of OB after treatment with a supernatant containing NETs, while the cell viability was restored after NETs were digested with DNase. In addition, some studies have found that NETs entrap MSU crystals, further stimulating NET accumulation of NETs around tophi [8, 9]. NETs are composed of an extracellular DNA network associated with polymorphonuclear neutrophil granule proteins, such as MPO and NE [10]. NE is an activity cluster of NETs and can hydrolyze various tissue proteins [24]. In the current study, the viability of OB was restored when NE was inhibited with sivelestat.

Multiple regression analysis showed that RANKL and OPG are independent factors of joint destruction in patients with gout [25], which suggests that the imbalance between OB and OC may cause joint injury through the OPG/RANKL/RANK pathway in gout [26]. OC are critical cells for local bone loss in gout [27]. Some studies found increased RANK mRNA and decreased OPG mRNA levels in the joints of patients with gout [7]. MSU crystals can promote neutrophils to adhere to OB [28]. RANKL is secreted by OB binding to the RANK receptor of OC precursor. The differentiation and maturation of OC lead to bone resorption [29]. In this study, we found decreased expression of ALP and OPG, and increased RANKL after treating with supernatant containing NETs. Once the NE was inhibited with sivelestat, the expression of markers was restored. This suggests that NE might induce imbalance between RANKL and OPG.

There are some limitations in the present study. The cytokines, chemokines, and proteases released by neutrophils may have a role in the pathology of bone erosion of gout [13]. Whether other factors influence the balance of OB/OC should be further explored. Also, more in vivo experiments of NE and the mechanisms between NE and the imbalance of RANKL/OPG are warranted.

## Conclusion

NETs induced by MSU crystals could inhibit osteoblasts viability and enhance the activity of osteoclasts. Moreover, NE might induce an imbalance between RANKL/OPG.

## Supplementary Information

**Additional file 1.**

## Data Availability

The datasets presented in this study can be found in online repositories: https://www.jianguoyun.com/p/DWrLpnMQlPGBChjtuMwEIAA.
