# Supplementary material for: Neutrophil extracellular traps induce the bone erosion of gout
Source: BMC Musculoskelet Disord. 2022 Dec 26;23:1128. doi: 10.1186/s12891-022-06115-w (PMC9791768; doi:10.1186/s12891-022-06115-w)
Supplement: Supplementary file 1 — Additional file 1. [file 12891_2022_6115_MOESM1_ESM.zip › supplement/Table S1.docx]

| H-ALP-F | GGACATGCAGTACGAGCTGA |
| --- | --- |
| H-ALP-R | GCAGTGAAGGGCTTCTTGTC |
| H-OPG-F | CAAAGTAAACGCAGAGAGTGTAGA |
| H-OPG-R | GAAGGTGAGGTTAGCATGTCC |
| H-RANKL-F | TCGATGGCTCATGGTTAGATC |
| H-RANKL-R | GGAACCAGATGGGATGTCGG |

**Table S1** lists the primers used in this study.
