# Supplementary figures and images for: Neutrophil extracellular traps induce the bone erosion of gout
Source: BMC Musculoskelet Disord. 2022 Dec 26;23:1128. doi: 10.1186/s12891-022-06115-w (PMC9791768; doi:10.1186/s12891-022-06115-w)

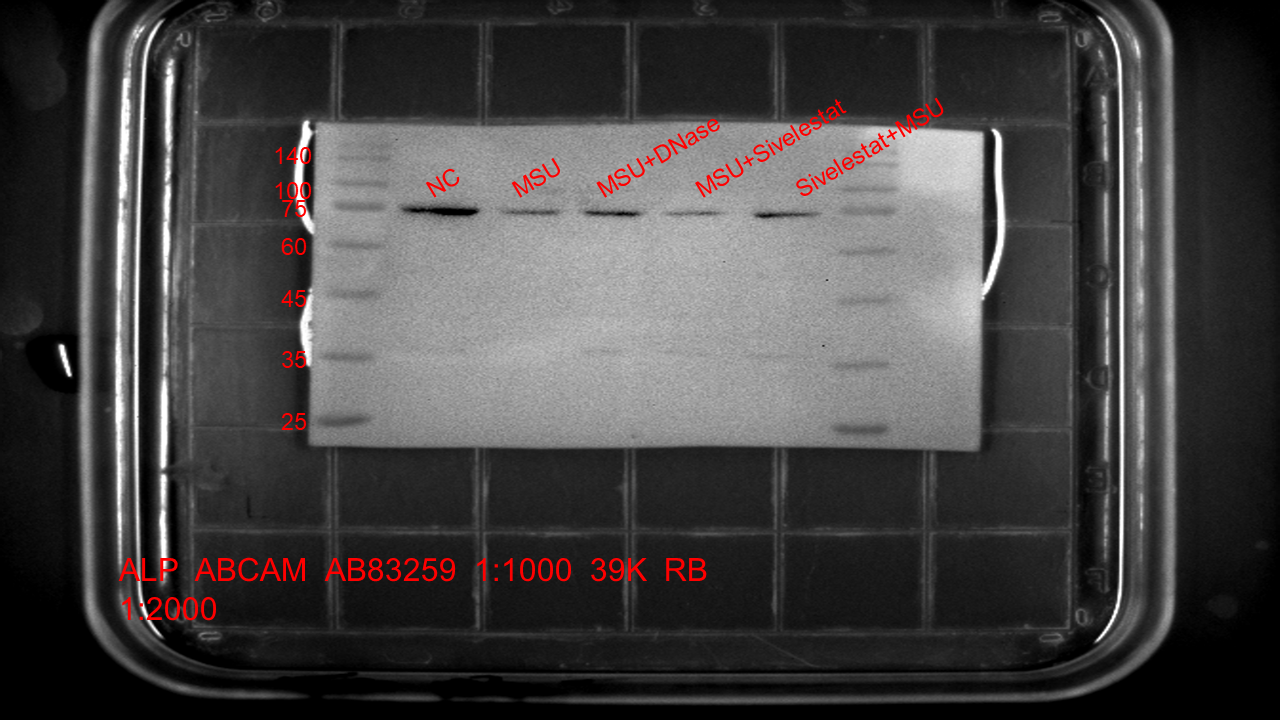

Supplement: Supplementary file 1 — Additional file 1. [file 12891_2022_6115_MOESM1_ESM.zip › supplement/gels and blots/fig 3d WB ALP.tif]

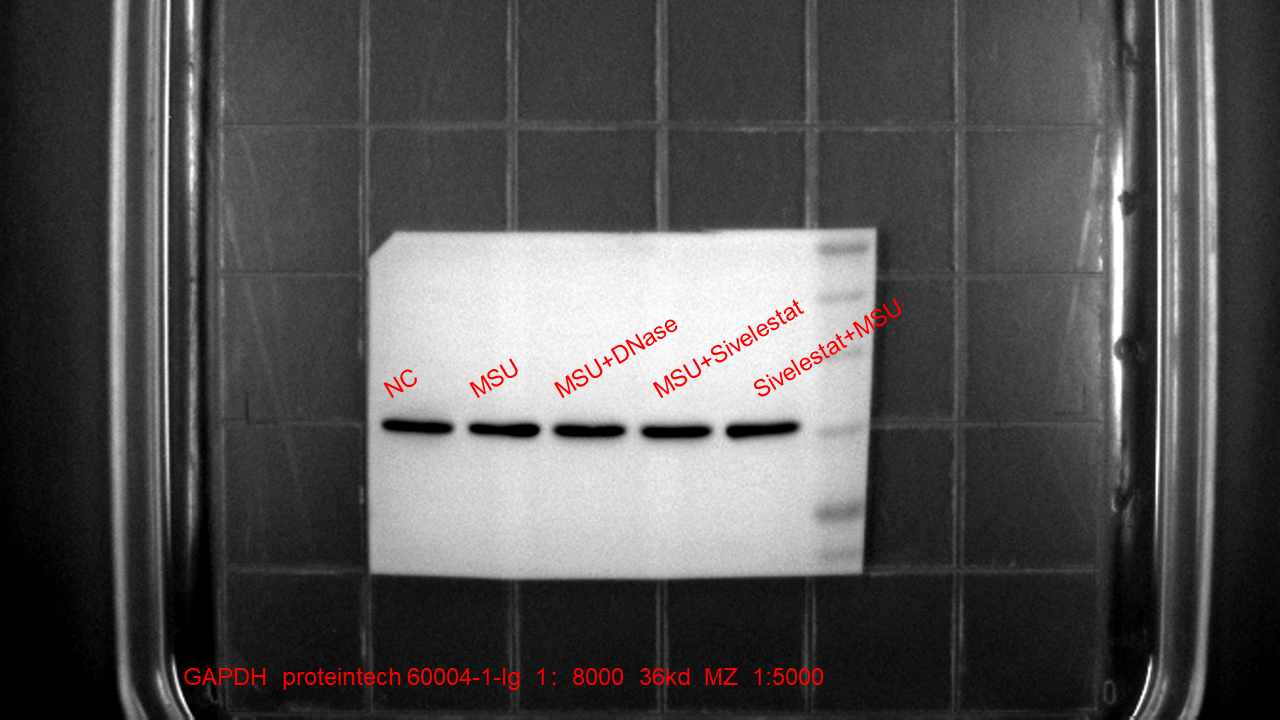

Supplement: Supplementary file 1 — Additional file 1. [file 12891_2022_6115_MOESM1_ESM.zip › supplement/gels and blots/fig 3d WB GAPDH.tif]

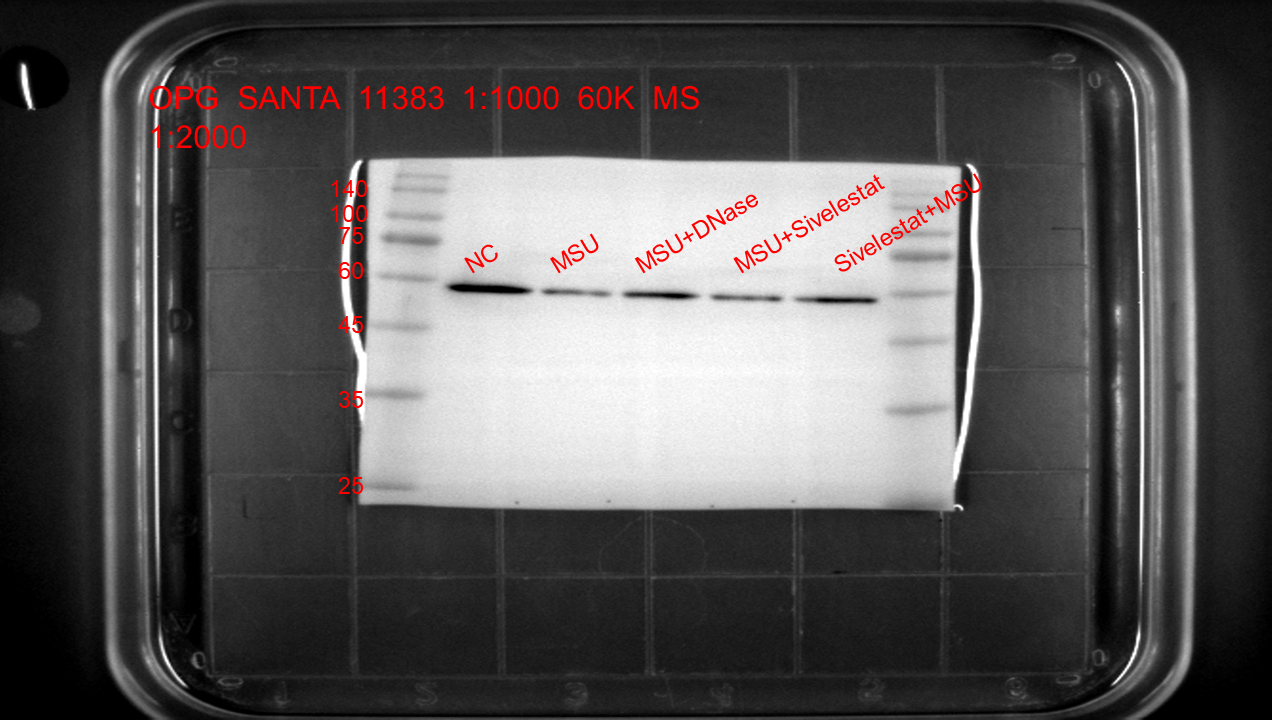

Supplement: Supplementary file 1 — Additional file 1. [file 12891_2022_6115_MOESM1_ESM.zip › supplement/gels and blots/fig 3d WB OPG.tif]

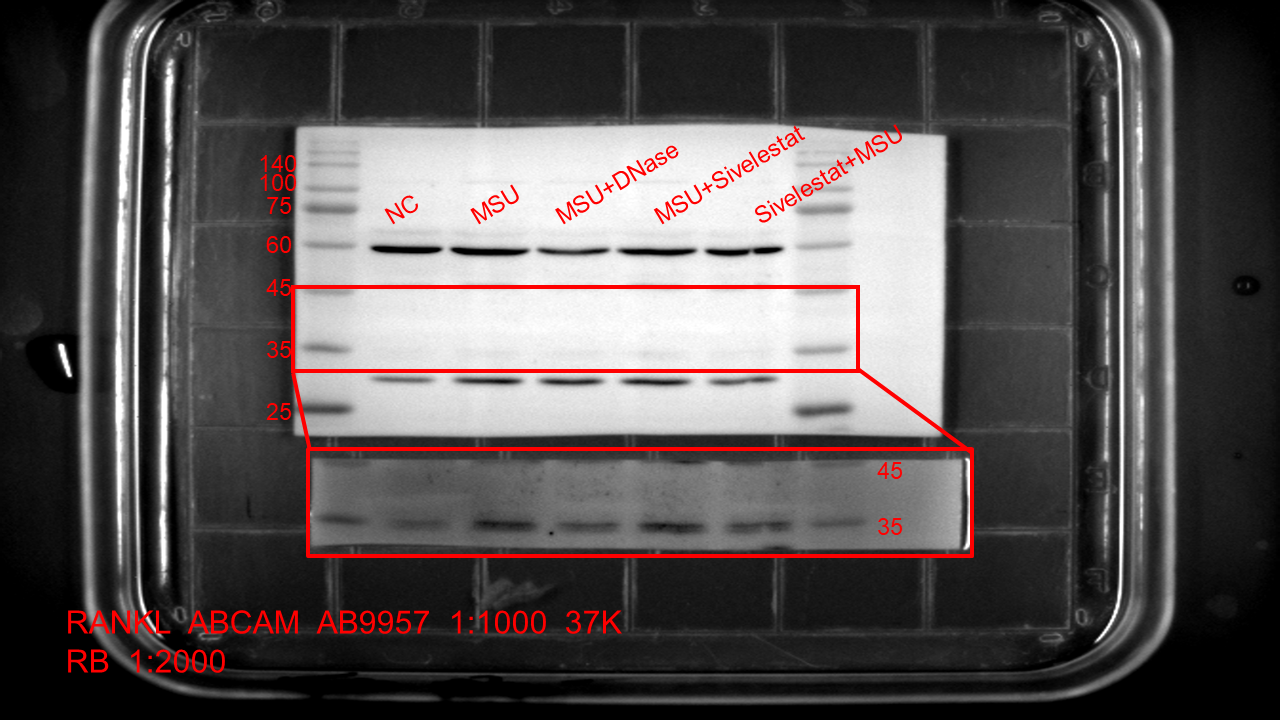

Supplement: Supplementary file 1 — Additional file 1. [file 12891_2022_6115_MOESM1_ESM.zip › supplement/gels and blots/fig 3d WB RANKL.tif]

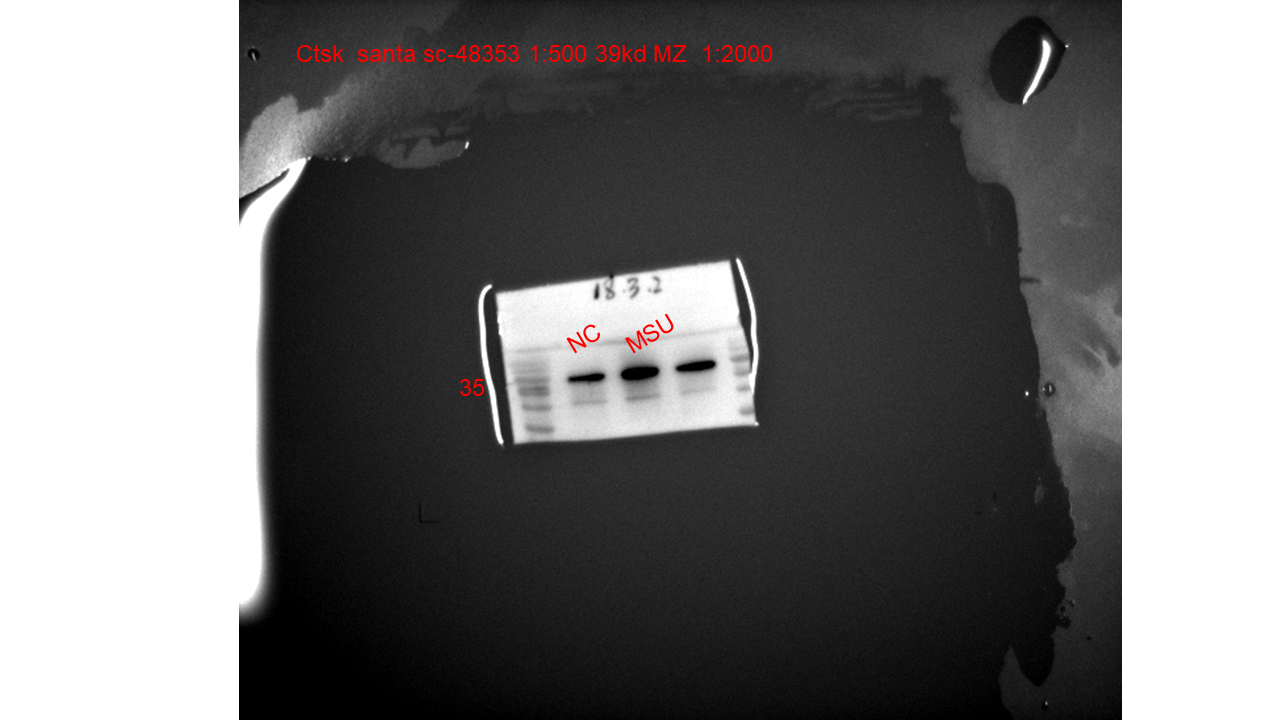

Supplement: Supplementary file 1 — Additional file 1. [file 12891_2022_6115_MOESM1_ESM.zip › supplement/gels and blots/fig 4a WB Ctsk.tif]

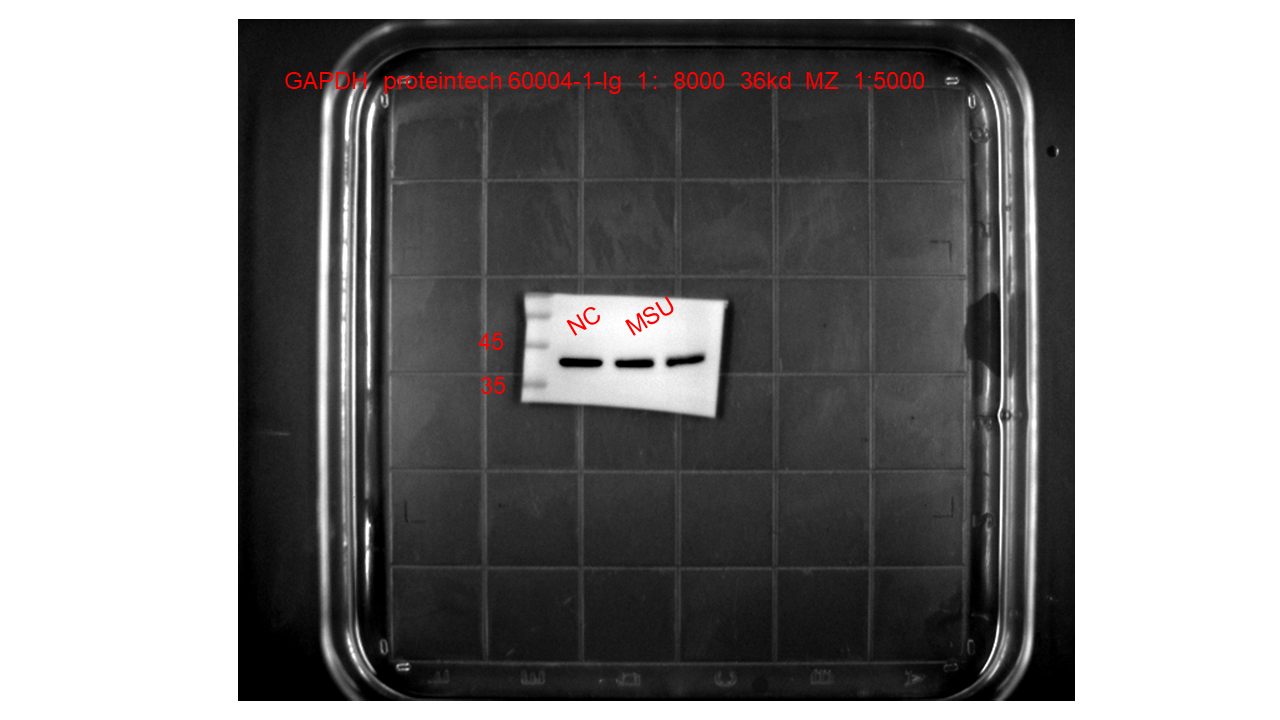

Supplement: Supplementary file 1 — Additional file 1. [file 12891_2022_6115_MOESM1_ESM.zip › supplement/gels and blots/fig 4a WB GAPDH.tif]

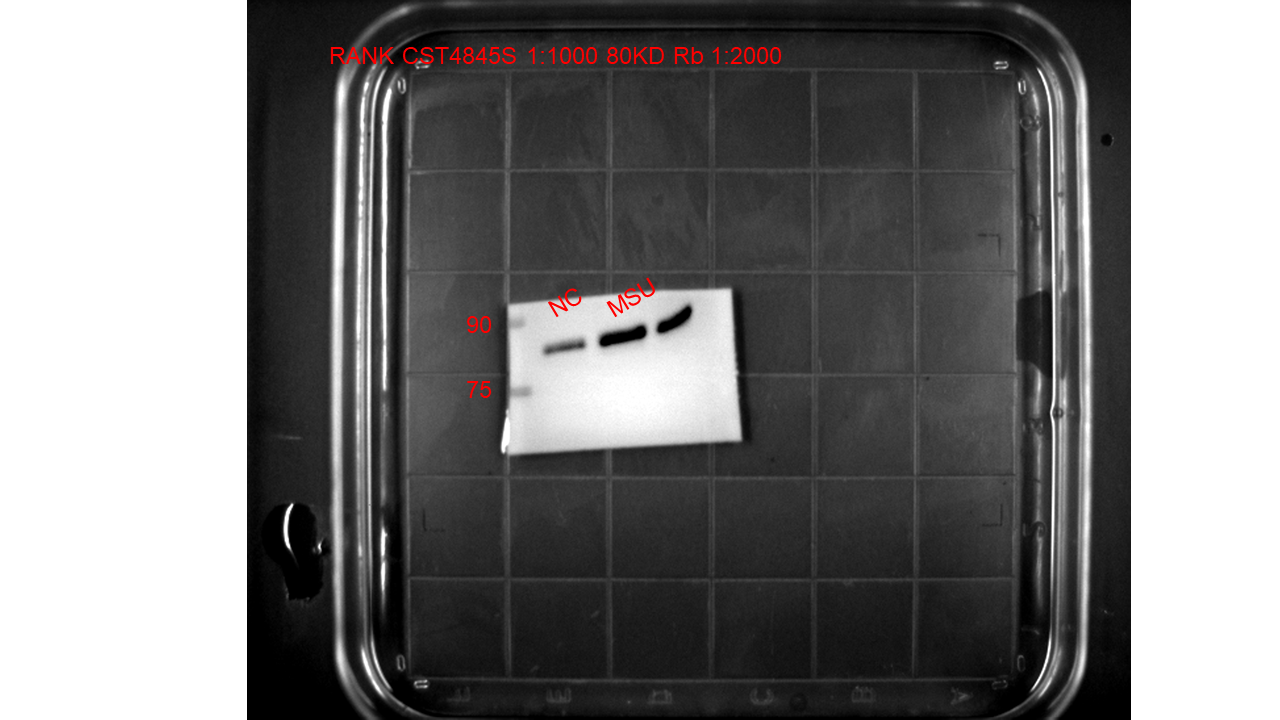

Supplement: Supplementary file 1 — Additional file 1. [file 12891_2022_6115_MOESM1_ESM.zip › supplement/gels and blots/fig 4a WB RANK.tif]

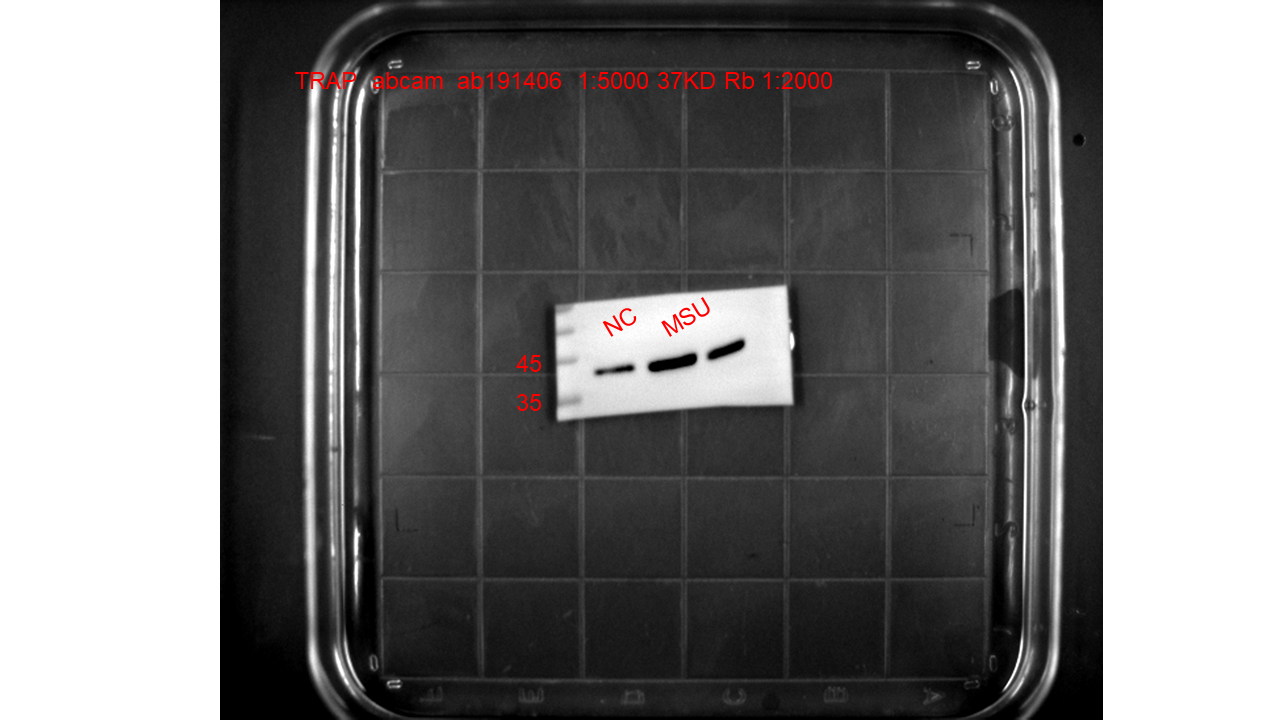

Supplement: Supplementary file 1 — Additional file 1. [file 12891_2022_6115_MOESM1_ESM.zip › supplement/gels and blots/fig 4a WB TRAP.tif]

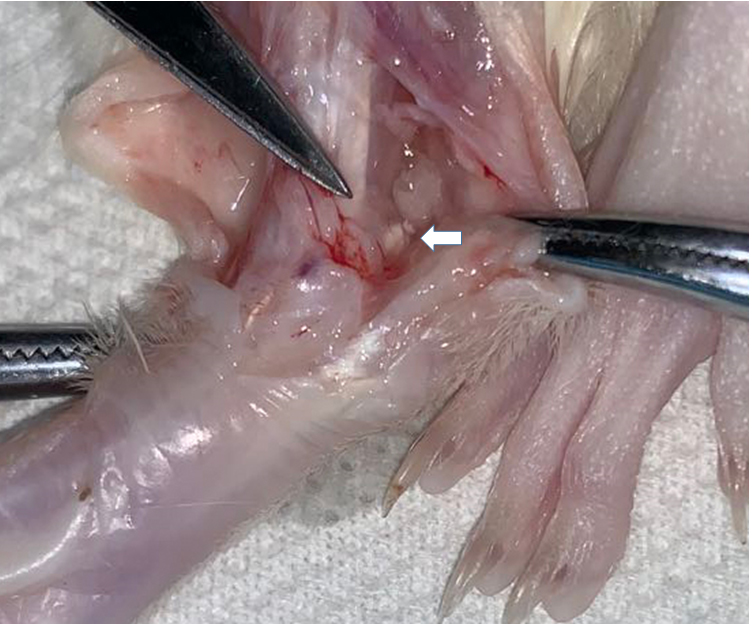


Legends

Supplement figure 1. Arrowheads point to the MSU deposits

Supplement: Supplementary file 1 — Additional file 1. [file 12891_2022_6115_MOESM1_ESM.zip › supplement/supplement fig 1.docx]
